# Category specificity in holistic processing: Reciprocal face–word interference does not extend to body stimuli

**DOI:** 10.3758/s13414-026-03270-3

**Published:** 2026-04-30

**Authors:** Paulo Ventura, Alexandre Pereira, Tina T. Liu

**Affiliations:** 1 CICPSI, Faculdade de Psicologia, Universidade de Lisboa, Alameda da Universidade, 1649-013 Lisbon, Portugal; 2 Centre for Research in Applied Communication, Culture and New Technologies (CICANT), Universidade Lusófona de Humanidades e Tecnologias, Lisbon, Portugal; 3 Universidade Europeia, Lisbon, Portugal; 4 Department of Neurology and Center for Brain Plasticity and Recovery, Georgetown University Medical Center, Washington, DC USA

**Keywords:** Faces, Words, Holistic processing, Interference, Bodies

## Abstract

This study examined the category specificity of a previously reported reciprocal interference between holistic face and word processing by incorporating body stimuli as a control. Prior work has demonstrated that superimposed faces and words disrupt each other’s holistic processing: faces are processed less holistically when paired with aligned words, and vice versa. Here, we asked whether a similar pattern of interference extends to body stimuli. In Experiment 1, we assessed whether face alignment would influence holistic body processing. We found no difference in holistic body processing between face-aligned and face-misaligned conditions, suggesting that faces do not interfere with holistic body processing. In Experiment 2, we examined whether word alignment affected body processing and again found no evidence of interference. Together, these findings indicate that body processing is unaffected by the alignment of overlaid words or faces, highlighting both the specificity and the limits of shared holistic processing mechanisms across high-level visual categories.

## Introduction

Whether word and face recognition rely on shared or distinct cognitive processes and neural resources has been the subject of extensive debate. Competing theoretical frameworks have emerged, with some advocating for the independence of these domains, while others propose overlapping processes (Burns & Bukach, 2021, 2022; Gerlach & Starrfelt, 2022; see Rossion & Lochy, 2021, for recent reviews). Human neuroimaging studies suggest that face and word processing rely on dissociable neural substrates within the ventral temporal cortex (VTC), which contains functionally specialized, category-selective regions such as the Fusiform Face Area (FFA) and the Visual Word Form Area (VWFA) (Cohen et al, 2000; Kanwisher et al., 1997). These findings support a modular view of cognitive architecture (Burns et al., 2017; Rubino et al., 2016; Saygin et al., 2015; Starrfelt et al., 2018; Susilo & Duchaine, 2013; Susilo et al., 2015). Further supporting this account, recent intracranial EEG evidence demonstrates that face- and word-selective responses within the VTC are dissociable, with distinct hemispheric and spatial distributions and minimal cross-category response correlations (Hagen et al., 2021).

However, alternative accounts challenge the modular view by emphasizing the interdependence and competition between face and word processing. According to the many-to-many hypothesis (Behrmann & Plaut, 2013, 2014, 2015, 2020; Plaut & Behrmann, 2011), the acquisition of literacy leads to cortical recycling in the ventral visual cortex. As individuals learn to read, neural representations of words increasingly rely on the left ventral cortex, which may, in turn, shift face processing to become more strongly lateralized to the right hemisphere (Behrmann & Plaut, 2015; Dehaene et al., 2015). From this distributed perspective, the systems supporting face and word recognition demonstrate overlapping and graded functional specialization both within and across hemispheres.

At the perceptual level, both face and word recognition require discrimination of visually similar exemplars. For faces, this challenge arises because all faces are composed of the same basic features organized in a common configuration (eyes above the nose; nose above the mouth). Successful face perception therefore requires fine-grained individuation beyond basic category recognition. Under the framework of perceptual expertise, similar demands have been observed in visual word recognition (e.g., Ventura, 2014; Wong & Gauthier, 2007). Skilled reading involves the rapid identification of a large number of highly similar words that are composed of a limited set of alphabetic symbols arranged in various combinations (Kleinschmidt & Cohen, 2006; Wong et al., 2011a).

**Fig. 1:**
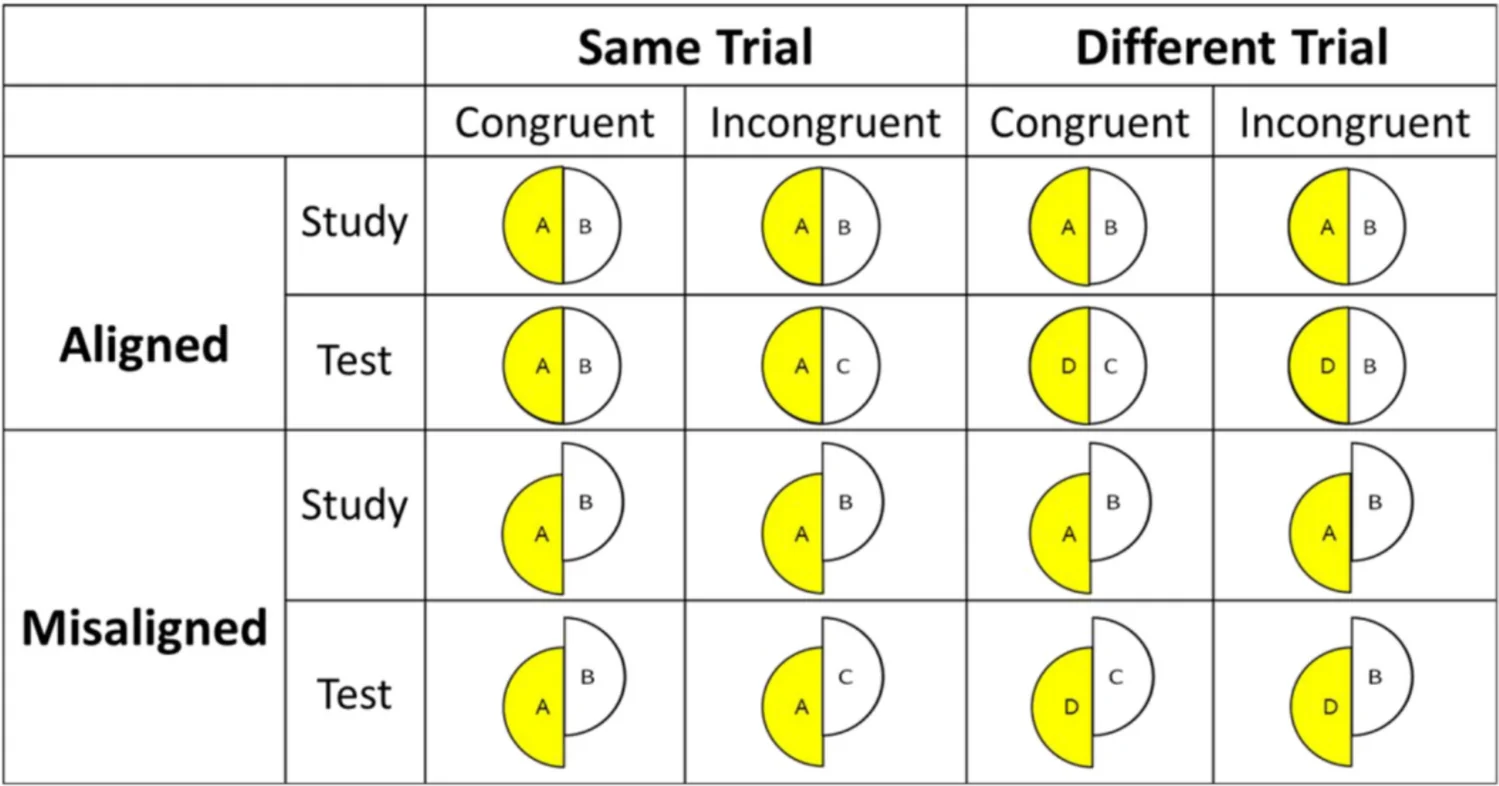
Illustration of the composite task using left-right face composites. Participants are instructed to attend to the target half (highlighted in yellow) while ignoring the other half. Figure adapted from Liu and Behrmann (2014)

A key mechanism underlying such perceptual expertise is holistic processing – the obligatory allocation of attention to an object as an integrated whole. Originally characterized in face perception (Diamond & Carey, 1986; Gauthier & Bukach, 2007; Gauthier et al., 2003; Rossion, 2013; Young et al., 1987), holistic processing has since been implicated in expert-level recognition across various domains, including words (e.g., Ventura et al., 2017, 2020; Wong et al., 2011b, 2012). In both domains, evidence for holistic processing has been mainly obtained using the composite task (Richler et al., 2012; Richler & Gauthier, 2014; Ventura et al., 2017, 2020; Wong et al., 2011b, 2012). In this paradigm, participants are asked to judge whether target parts (e.g., the left halves of two faces) of two consecutively presented stimuli are the same or different while ignoring the irrelevant parts (e.g., the right halves of two faces; Fig. 1). Despite being instructed to focus solely on the relevant parts, irrelevant parts nevertheless affect performance. When irrelevant parts lead to a congruent response with the relevant part (i.e., “same” or “different” for both the target and distractor parts), performance is enhanced. Conversely, this congruency effect is often diminished when the target and distractor parts are misaligned, which disrupts configural face processing.

Building on this framework, our recent study (Ventura et al., 2023) tested whether the holistic processing of faces and words interferes with each other when both are present. Using a modified composite task (Curby & Moerel, 2019), we superimposed faces and words and instructed participants to selectively attend to one category. We found a bi-directional trade-off: faces were processed less holistically when superimposed with aligned words, and vice versa, suggesting that word and face recognition rely on partially shared cognitive processes and cortical resources.

To evaluate whether this trade-off is specific to faces and words – both categories contain many visually confusable and homogeneous exemplars, and are thought to rely on high-acuity regions of visual cortex (Hasson et al., 2002; Levy et al., 2001) – we included body stimuli as a control condition. In Experiment 1, we tested interference between bodies and faces; in Experiment 2, we tested interference between bodies and words. If holistic processing of faces and bodies, or of words and bodies, relies on shared mechanisms, we would expect similar interference when superimposed stimuli are aligned. Alternatively, observing minimal interference would suggest greater functional independence between these domains.

## Experiment 1: Impact of unattended faces on holistic body processing

To test whether the category-specific interference observed between faces and words extends to other domains, Experiment 1 examined the influence of unattended faces on the processing of another category known to be processed holistically: bodies. Participants were instructed to attend to bodies while ignoring superimposed faces.

The within-subject factors were body alignment (aligned vs. misaligned), body congruency (congruent vs. incongruent), and face alignment (aligned vs. misaligned). Holistic processing of the attended category (bodies) was assessed via an interaction between body alignment and body congruency – a standard index of holistic processing in the context of the composite task.

The primary question of interest was whether task-irrelevant face alignment interferes with holistic body processing. Such interference would be reflected in a three-way interaction among face alignment, body alignment, and body congruency. Holistic body processing was quantified using the subtraction: (congruent aligned body – incongruent aligned body) - (congruent misaligned body – incongruent misaligned body). The critical comparison was the magnitude of holistic body processing across face-aligned and face-misaligned conditions, indicating whether irrelevant face alignment influences body processing.

### Method

#### Participants

Prior to the study, we performed a power analysis based on results from Curby and Moerel (2019). Specifically, we leveraged the critical three-way interaction between line pattern alignment, face alignment, and face congruency (η_p_^2^ =.26) in Experiment 1 of Curby and Moerel (2019). Using MorePower 6.0.4 (Campbell & Thompson, 2012), a sample size of 26 would be required to detect a comparable effect at α = 0.05 with power of 0.8 for a 2 × 2 × 2 repeated-measures ANOVA.

A total of 34 participants took part in the experiment. The study’s protocol adhered to the guidelines of the Declaration of Helsinki and the Portuguese deontological regulation for psychology and was approved by the Deontological Committee of the Faculdade de Psicologia of the Universidade de Lisboa. All participants provided informed consent prior to participation.

#### Stimuli

##### Composite faces

We used matched left-right face composites, drawn from the Caucasian face subset used in the Liu and Behrmann (2014) and Liu et al. (2014) studies. Twenty faces were subdivided into five groups of four similar faces based on prior, independently assessed ratings. This ensured that the task could not be performed based purely on low-level facial symmetry cues. Each aligned composite face was then created by pairing the left half of one face with the right half of another face from the same group (274 × 384 pixels). Each misaligned composite face was created by moving the right half down by approximately one-third of the face (274 × 464 pixels).

##### Composite bodies

Matched left-right body composites were created using 20 body images selected from Stigliani et al. (2015). The 20 bodies were subdivided into five groups of four similar bodies based on prior, independently collected ratings. This ensured that the task could not be performed based purely on body symmetry cues. Each aligned composite body was then created by pairing the left half of one body with the right half of another body from the same group (276 × 390 pixels). Each misaligned composite body was created by moving the right half down by approximately one-third of the body (276 × 470 pixels).

#### Procedure

**Fig. 2:**
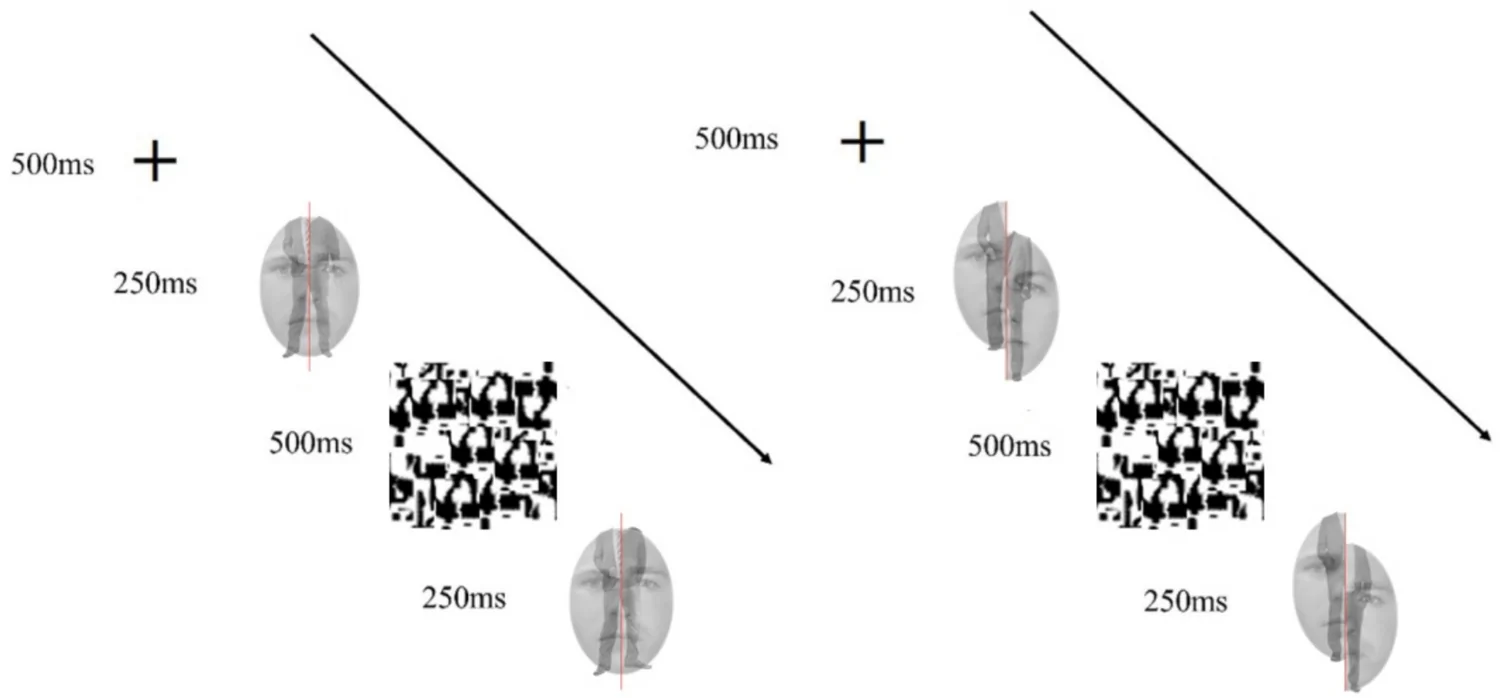
Procedure used for the modified body-face composite task. Composite stimuli were created by pairing aligned or misaligned faces with aligned or misaligned bodies. Left: Example of an aligned body and aligned face trial. Right: Example of a misaligned body and misaligned face trial

Each participant completed a total of 384 trials divided across four blocks. The experiment was programmed in E-prime Go (https://pstnet.com/eprime-go/) and was run entirely online, with participants completing the study at home. On each trial, body composite images were presented with face composite images overlaid on top (see Fig. 2). A 2-pixel-wide vertical red line was placed on the midline of each face and the midline of each body. Each trial proceeded as follows (see Fig. 2):

1. Fixation screen (500 ms),
2. Study stimulus (i.e., a body composite with a face composite overlaid; 250 ms),
3. Pattern mask (500 ms),
4. Test stimulus (i.e., a body composite with a face composite overlaid; 250 ms).

Participants were instructed to make same-different judgments on the left half of the two sequentially displayed bodies while ignoring the right half of the bodies and the overlaid faces. Each participant completed 16 practice trials to familiarize themselves with the task prior to the experiment.

The left and right halves of face and body stimuli were either aligned or misaligned, resulting in four stimulus configurations (“face aligned, body aligned,” “face aligned, body misaligned,” “face misaligned, body aligned,” and “face misaligned, body misaligned”). Trials with the same stimulus configurations were blocked, and the block order was randomized across participants (96 trials per block). The correct response for the left half of the body image (same or different) and the congruency between the task-irrelevant right halves were counterbalanced within each block. The congruency for the task-irrelevant faces was also counterbalanced with respect to body congruency within each block.

### Results

We report both accuracy and response time (RT) across the two experiments. To control for multiple comparisons, a Bonferroni correction was applied to the planned *t*-tests. Given four comparisons, the adjusted significance threshold was set to α = 0.0125 (0.05/4).

#### Accuracy

A 2 (body alignment: aligned, misaligned) × 2 (body congruency: congruent, incongruent) × 2 (face alignment: aligned, misaligned) ANOVA was performed. If faces and bodies engage shared holistic processing, we would expect a trade-off in holistic processing when they are superimposed and aligned. Bodies would be processed less holistically when superimposed with aligned faces than with misaligned faces because aligned faces are also processed holistically. Alternatively, if holistic processing of faces and bodies is relatively independent (i.e., can occur in parallel with minimal interference), then we would expect little difference in holistic body processing when face stimuli are aligned or misaligned.

The ANOVA performed on the accuracy scores revealed no significant main effects or interactions, including the three-way interaction, *F*(1, 32) = 2.81, *p* =.10, ηp2 =.08. The interaction of body alignment × body congruency, *F* < 1was not significant.

**Fig. 3:**
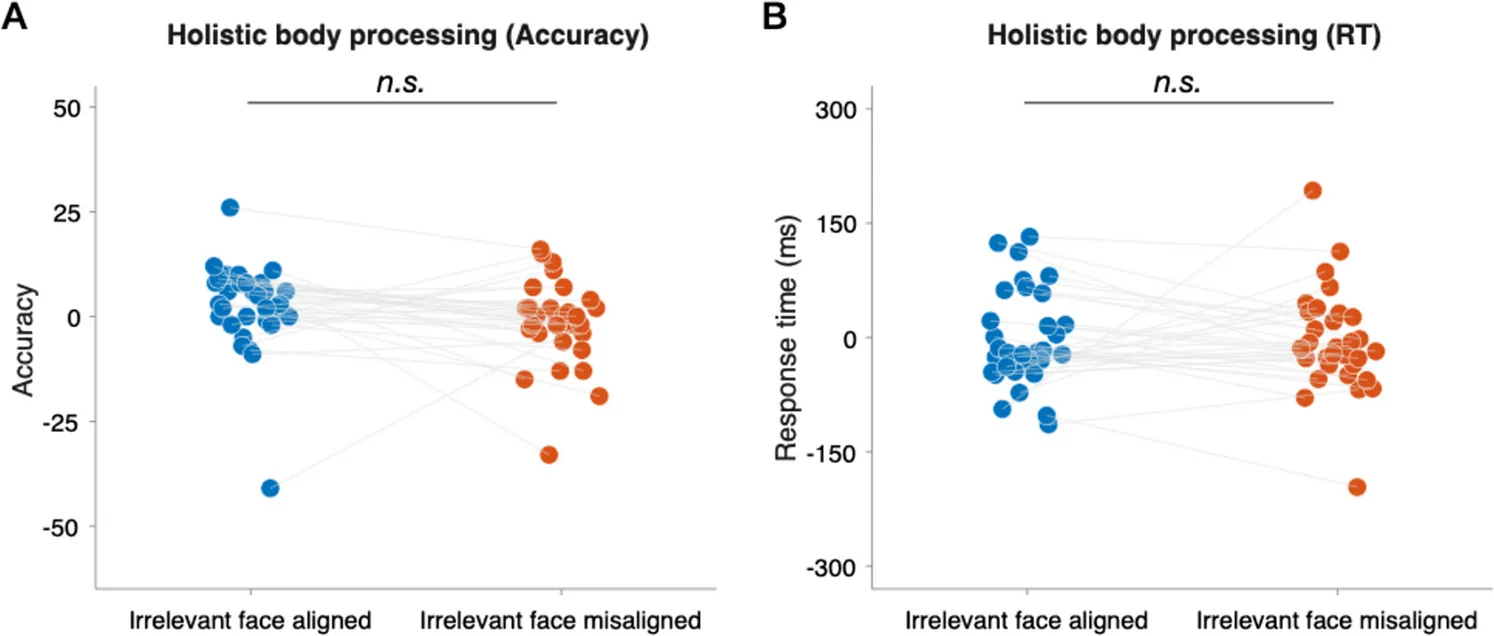
Holistic body processing as a function of face alignment. Body accuracy (**A**) and response time (RT) (**B**) are shown for conditions in which the task-irrelevant face was either aligned or misaligned

Although the body composite effect was not significant, it was still relevant to compare the magnitude of holistic body processing when irrelevant faces were aligned or misaligned. Holistic body processing was numerically smaller in the presence of misaligned faces (mean = −1.4242, *SD* =.10) than in the presence of aligned faces, (mean = 2.7273, *SD* =.10), but the difference was not statistically significant, *t*(32) = 1.68, *p* =.10 (Fig. 3A).

We performed additional Bayesian analyses to compare evidence for the null versus the alternative hypothesis. Bayesian inference incorporates prior information and quantifies how much more likely the observed data are under the null hypothesis compared to the alternative hypothesis. Specifically, the Bayes factor (BF01) represents the likelihood of the data under the null hypothesis (no condition difference) relative to the alternative (a condition difference) and is directly interpretable as an odds ratio. This approach allows us to assess whether there is sufficient evidence in favor of either hypothesis.

We compared the null and alternative hypotheses regarding the magnitude of holistic body processing across face-aligned and face-misaligned conditions to determine whether irrelevant face alignment influences body processing. Bayes factors (BF01) were computed using JASP (version 0.95.4) with default prior distributions (e.g., for Student’s *t*-test), appropriate when prior knowledge is absent or vague. A Bayes factor BF01 quantifies the evidence in favor of the null hypothesis (*H*0) relative to the alternative hypothesis (*H*1). Values greater than 1 indicate support for the null hypothesis, whereas values less than 1 indicate support for the alternative hypothesis. BF01 values are interpreted as follows:

**Table:** 

*BF₀₁ value*	*Interpretation*
> 100	Extreme evidence for H₀
30–100	Very strong evidence for H₀
10–30	Strong evidence for H₀
3–10	Moderate evidence for H₀
1–3	Anecdotal (weak) evidence for H₀
≈ 1	No evidence; data are about equally likely under both hypotheses
1/3–1	Anecdotal evidence for H₁
1/10–1/3	Moderate evidence for H₁
1/30–1/10	Strong evidence for H₁
1/100–1/30	Very strong evidence for H₁
< 1/100	Extreme evidence for H₁

We obtained BF_01_ = 1.523, providing anecdotal support for the null hypothesis

#### Response times (RTs)

Trials with RTs < 200 ms or > 1,750 ms (similar to the criteria used in Curby & Moerel, 2019) were removed from the analysis (< 1%). Only trials with a correct response were analyzed. A 2 (body alignment: aligned, misaligned) × 2 (body congruency: congruent, incongruent) × 2 (face alignment: aligned, misaligned) ANOVA was performed on the RTs. If faces and bodies engage shared holistic processing, we would expect a trade-off in holistic processing. Bodies would be processed less holistically when superimposed with aligned than misaligned faces because aligned faces are also processed holistically. Alternatively, if holistic processing of faces and bodies is relatively independent (i.e., can be processed in parallel with less interference), then we would expect minimal differences in holistic body processing when face stimuli are aligned or misaligned.

The ANOVA revealed no significant main effects or interactions, including the three-way interaction, all *F*s < 1. The interaction of body alignment × body congruency, *F* < 1, was not significant.

We also compared RTs for holistic body processing as a function of face alignment (aligned vs. misaligned). The difference was not significant, *t*(32) =.21, *p* =.83 (Fig. 3B); BF_01_= 5.259, providing substantial support for the null hypothesis. In sum, across both accuracy and RT measures, we found no evidence that task-irrelevant face alignment interfered with holistic body processing, suggesting that holistic body processing operates independently of face alignment or processing.

## Experiment 2: Impact of unattended words on holistic body processing

To determine whether the pattern of interference observed with faces extends to another category, Experiment 2 examined whether unattended words affect holistic processing of bodies. Participants attended to bodies while ignoring superimposed words, which were presented in aligned or misaligned configurations to create high- and low-interference conditions, respectively.

The within-subject factors were body alignment, body congruency, and word alignment. Holistic body processing was reflected by the standard body alignment × body congruency interaction. The question of interest was whether task-irrelevant word alignment disrupts holistic body processing, which would be revealed by a three-way interaction among word alignment, body alignment, and body congruency. To further explore the source of any potential three-way interaction, trials with aligned and misaligned words were analyzed separately. The primary question was whether task-irrelevant word alignment interferes with holistic body processing. Thus, the critical comparison was the magnitude of holistic body processing across word-aligned and word-misaligned conditions.

### Method

#### Participants

Thirty-nine participants participated in this experiment.

#### Stimuli

##### Composite words

**Fig. 4:**
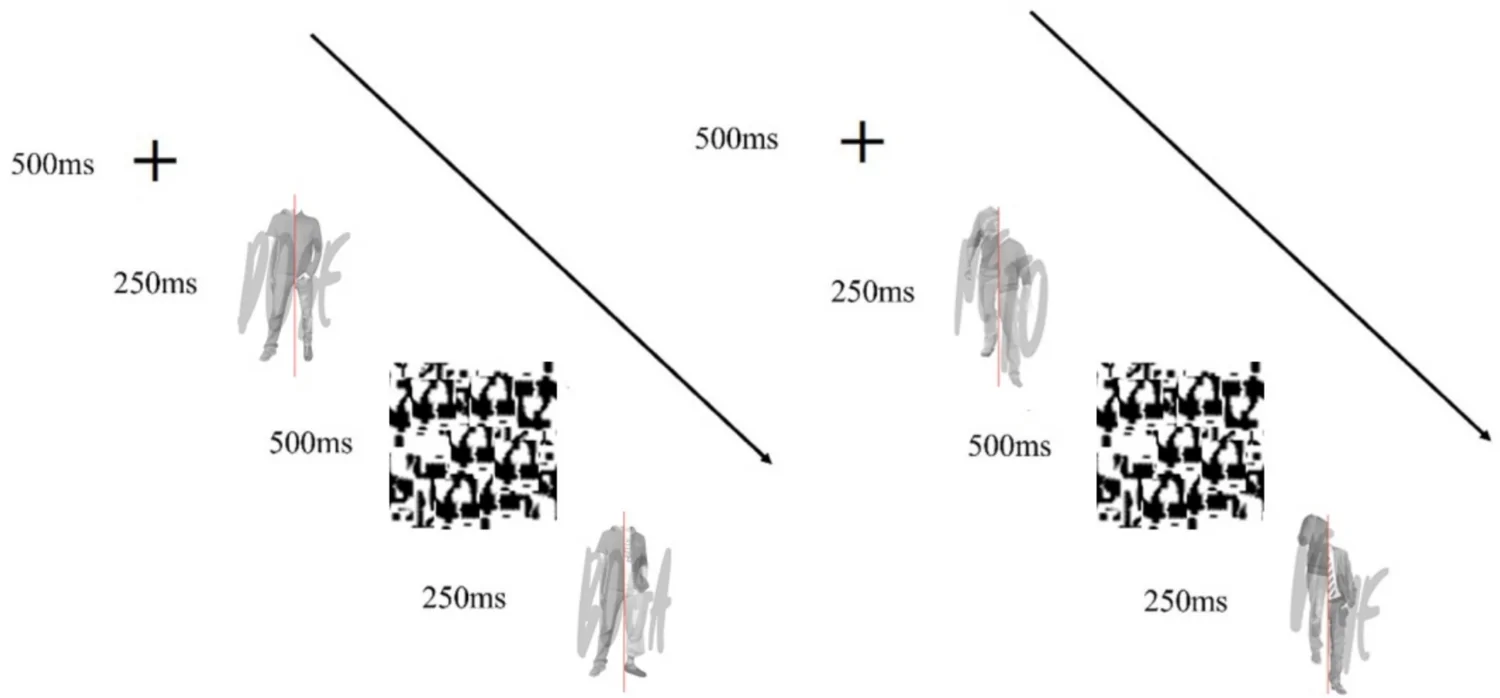
Procedure used for the modified body-word composite task. Composite stimuli were created by pairing aligned or misaligned words with aligned or misaligned bodies. **Left:** Example of an aligned body and aligned word trial. **Right:** Example of a misaligned body and misaligned word trial

A total of 168 disyllabic consonant-vowel.consonant-vowel (CV.CV) Portuguese words in Tracker font were used (see Fig. 4). A thin vertical red line (2-pixels wide) between the second and third letters divided each word into a left and a right half. Misaligned words were created by moving down the right half of the words by approximately 80 pixels on average (average word-aligned: 242 × 184 pixels; average word-misaligned: 242 × 264 pixels).

##### Composite bodies

These were the same as in Experiment 1.

#### Procedure

Each participant completed a total of 384 trials divided over four blocks. The experiment was programmed in E-prime Go (https://pstnet.com/eprime-go/) and participants completed the study at home. In each trial, body composite images were presented with word composite images overlaid (see Fig. 4). A 2-pixel-wide vertical red line was placed on the midline of each word and the midline of each body. Each trial proceeded as follows (see Fig. 4): (1) fixation screen (500 ms), (2) study stimulus (i.e., a body composite with a word composite overlaid; 250 ms), (3) pattern mask (500 ms), (4) test stimulus (i.e., a body composite with a word composite overlaid; 250 ms). Participants were instructed to make same-different judgments on the left half of the two sequentially displayed bodies while ignoring the right half of the bodies and the overlaid words. Each participant completed 16 practice trials to familiarize themselves with the task prior to the experiment.

### Results

#### Accuracy

A 2 (body alignment: aligned, misaligned) × 2 (body congruency: congruent, incongruent) × 2 (word alignment: aligned, misaligned) ANOVA performed on the accuracy scores revealed a three-way interaction among word alignment, body alignment and body congruency, *F*(1, 38) = 5.4, *p* =.025, ηp2 =.12. To investigate the underlying source of this three-way interaction, the data from the trials where the words were aligned and those where they were misaligned were analyzed separately. Within both the aligned word condition and the misaligned word condition the 2 (body congruency) × 2 (body alignment) ANOVA revealed an interaction between body congruency and body alignment, *F*(1, 38) = 54.63, *p* <.0001, ηp2 =.59; *F*(1, 38) = 17.7, *p* <.00015, ηp2 =.32, respectively. In both cases, the congruency effect was higher in the misaligned body condition than in the aligned body condition, which is counter to the usual direction of the composite effect.

**Fig. 5:**
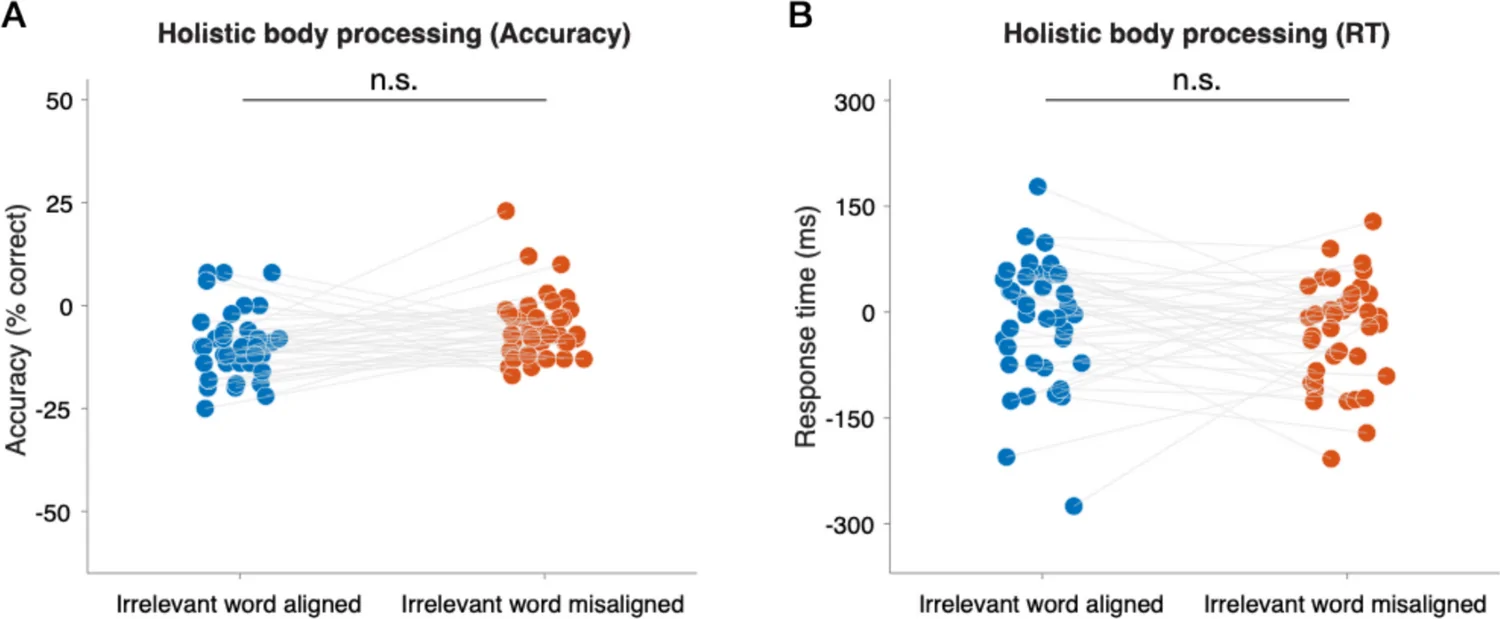
Holistic body processing as a function of word alignment. Body accuracy (**A**) and response time (RT) (**B**) are shown for conditions in which the task-irrelevant word was either aligned or misaligned

We also compared the magnitude of holistic body processing when irrelevant words were aligned or misaligned. Holistic body processing was not significantly higher in the presence of misaligned words (mean = −5.3333, *SD* =.08) than in the presence of aligned words (mean = −9.7179, *SD* =.08), *t*(38) = 2.33, *p* =.025; *p* >.0125 (Bonferroni-corrected comparison) (Fig. 5A), BF_01_ = 0.533. The Bayes factor indicates anecdotal support for the alternative hypothesis. In sum, we found no difference between holistic body processing under word-aligned versus word-misaligned conditions, suggesting that task-irrelevant word alignment does not interfere with holistic body processing.

#### RTs

Trials with RT < 200 ms or > 1,750 ms (similar to the criteria used in Curby & Moerel, 2019) were removed from the analysis (< 1%). Only trials with a correct response were analyzed.

The RT analysis revealed a significant effect of body congruency, *F*(1, 38) = 5.40, *p* =.026, ηp2 =.12, and a significant interaction of body alignment × body congruency, *F*(1, 38) = 4.22, *p* =.047, ηp2 =.10, showing evidence of a composite body effect with a higher congruency effect for body-aligned than body-misaligned conditions; magnitude of the effect = 0.15. The body composite effect is numerically smaller than the effects obtained in a similar paradigm in Ventura et al. (2023): face composite effect: 0.20; word composite effect: 0.32. See a summary of face, word, and body composite effects in Table 1. The ANOVA performed on the RTs revealed no other significant effects, including the three-way interaction, *F* < 1.

**Table 1:** Magnitude of face and word composite effects in Ventura et al. (2023) and body composite effects observed in the present study

Publication	Type of composite effect	Magnitude of composite effect
Ventura et al. (2023)	Face composite effect	0,20 (sensitivity)
Ventura et al. (2023)	Face composite effect	0,10 (RT)
Ventura et al. (2023)	Word composite effect	0,03 (sensitivity)
Ventura et al. (2023)	Word composite effect	0,32 (RT)
Present study (Experiment 1)	Body composite effect	0,10 (sensitivity)
Present study (Experiment 1)	Body composite effect	0,09 (RT)
Present study (Experiment 2)	Body composite effect	0,05 (sensitivity)
Present study (Experiment 2)	Body composite effect	0,15 (RT)

The comparison of the magnitude of holistic body processing when irrelevant words were aligned or misaligned was not significant, *t*(28) =.88, *p* =.39; BF_01_= 4.043, indicating substantial support for the null hypothesis (Fig. 5B).

## General discussion

The goal of this study was to evaluate the specificity of a previously reported reciprocal interference between holistic face and word processing by introducing body stimuli as a control. In earlier work (Ventura et al., 2023), we found that faces and words disrupt each other’s holistic processing when superimposed – faces were processed less holistically when paired with aligned words, and vice versa. This bi-directional trade-off challenges strictly modular accounts of category-selective processing and suggests that face and word processing rely on partly shared cognitive resources, likely shaped by perceptual expertise and the demands of individuating highly confusable exemplars (Kleinschmidt & Cohen, 2006; Richler & Gauthier, 2014; Wong & Gauthier, 2007).

The current study extends these findings by testing whether similar interference arises with body stimuli. The human body is a primary social stimulus that conveys crucial information for identity recognition (Yovel & O’Toole, 2016) and social interaction (de Gelder, 2009). Like faces, bodies are processed holistically (Butti et al., 2022; Harris et al., 2016; Reed et al., 2015; Willems et al., 2014), and their perception recruits the fusiform body area (FBA; Downing et al., 2001; Peelen & Downing, 2005), which is anatomically adjacent to but functionally distinct from the fusiform face area (FFA; Schwarzlose et al., 2005). In line with this neural dissociation, Experiment 1 revealed no difference in holistic body processing when faces were aligned versus misaligned, suggesting that face processing does not interfere with holistic body processing.

Similarly, in Experiment 2, word alignment did not disrupt holistic body processing – there were no differences in holistic body processing under word-aligned and word-misaligned conditions. Together, these results suggest that the interference observed between faces and words is specific to those domains and does not generalize to bodies. These findings align with the many-to-many hypothesis (Behrmann & Plaut, 2013, 2014, 2015, 2020), which proposes a distributed, interactive system in which face and word recognition involve overlapping but graded functional networks across and within hemispheres. The two experiments in this article demonstrate the category specificity of faces and words by showing that they are impervious to the influence of holistic body processing. Bodies do not seem to involve overlapping resources with either faces or words. Across the two experiments, for accuracy, the Bayes factor (BF_01_) indicated inconclusive evidence with respect to H_0_ versus H_1_, whereas for RT, the Bayes factor provided substantial evidence in favor of H_0_. Taken together, this pattern of results suggests no meaningful differences in holistic body processing between face-aligned and face-misaligned conditions, nor between word-aligned and word-misaligned conditions. Rather than merely being inconclusive, the data actively provide evidence supporting the absence of meaningful differences in holistic body processing under the influence of face alignment or word alignment.

The specificity of the reciprocal interference between holistic face and word processing may reflect the unique functional pressures associated with face and word processing. Both categories demand rapid, fine-grained discrimination among many visually similar exemplars and are supported by high-acuity representations in ventral visual cortex (Hasson et al., 2002; Levy et al., 2001). Neuroanatomically, face and word processing are associated with the FFA (Kanwisher et al., 1997) and the visual word form area (VWFA; Cohen et al., 2000), which occupy adjacent territory along the fusiform gyrus. Although these regions are lateralized – with the FFA typically in the right hemisphere and the VWFA in the left – functional and developmental evidence suggests some degree of competition or resource sharing between them (Behrmann & Plaut, 2015; Dehaene et al., 2015; Liu et al., 2018).

If interference effects between faces and words reflected a general feature of holistic processing or perceptual expertise, then similar trade-offs would be expected when bodies are superimposed with either faces or words. However, our findings provide no evidence for such interference. Instead, they suggest that holistic body processing operates independently of the mechanisms supporting holistic face and word processing, reflecting the fact that body perception recruits the fusiform body area (Downing et al., 2001; Peelen & Downing, 2005), which is anatomically adjacent to but functionally distinct from the FFA (Schwarzlose et al., 2005) and the nonadjacency between the FBA and the VWFA. Additionally, our results indicate that although bodies are processed holistically (cf. the RT results in Experiment 2 of this study), they do not interact with the holistic processing of either faces or words. According to the many-to-many hypothesis (Behrmann & Plaut, 2013, 2014, 2015, 2020; Plaut & Behrmann, 2011), the acquisition of literacy leads to cortical recycling in the ventral visual cortex. As individuals learn to read, neural representations of words increasingly rely on the left ventral cortex, which may, in turn, shift face processing to become more lateralized to the right hemisphere (Behrmann & Plaut, 2015; Dehaene et al., 2015; Liu et al., 2018). Thus, the interaction appears to be between words and faces and should not extend to other categories like bodies. Importantly, we found evidence of holistic body processing in the RT data of Experiment 2, although in the accuracy data of Experiment 2 the direction of the effect was contrary to a composite effect, and we found no interaction with word (mis)alignment. Previous studies have reported holistic processing using the RT measure, but no significant holistic processing in sensitivity/accuracy analysis, thus this pattern is not unusual. In fact, it was similar to Experiment 2 of Curby and Moerel (2019).

In conclusion, across two experiments, we found no reciprocal interference between holistic body processing and either face or word alignment. These results support the view that face and word processing share specialized and overlapping resources that do not extend to other socially meaningful categories like bodies. This highlights both the specificity and the limits of shared processing mechanisms in high-level vision.

## Data Availability

The data for all experiments are available at https://osf.io/mx64z/?view_only=058bbead56324f038685e3c819b3a1fb. Materials for the experiments are available upon request.

## Code availability

Eprime program code is available upon request
